# Supplementary material for: Neurological Involvement in Children with COVID-19 and MIS-C: A Retrospective Study Conducted for More than Two Years in a Pediatric Hospital
Source: Children (Basel). 2022 Nov 24;9(12):1809. doi: 10.3390/children9121809 (PMC9777384; doi:10.3390/children9121809)
Supplement: Supplementary file 1 [file children-09-01809-s001.zip › children-2020104-supplementary.pdf]

Table S1. Neurological manifestations at the onset in the enrolled patients and in the two subgroups.

**Neurological Manifestations**	**Total (*n* = 122)**	**COVID-19 (*n* = 95)**	**MIS-C (*n* = 27)**	***p***
Total, *n* (%)	70 (57.4)	58 (61.1)	12 (44.4)	0.124
Time between disease onset and neurological manifestations, median (IQR) – *days*	0 (0-2)	0 (0-1)	2 (0-3)	0.007
Consciousness impairment, *n* (%)	36 (29.5)	34 (35.8)	2 (7.4)	0.004
Irritability/agitation, *n* (%)	21 (17.2)	20 (21.1)	1 (3.7)	0.042
Drowsiness/hyporeactivity, n (%)	17 (13.9)	16 (16.8)	1 (3.7)	0.116
Confusion, *n* (%)	2 (1.6)	2 (2.1)	0	1.000
Temporary LOC, *n* (%)	2 (1.6)	2 (2.1)	0	1.000
Headache, *n* (%)	20 (16.4)	12 (12.6)	8 (29.6)	0.035
Seizures, *n* (%)	10 (8.2)	10 (10.5)	0	0.115
Dizziness, *n* (%)	2 (1.6)	1 (1.1)	1 (3.7)	0.395
Dysgeusia, *n* (%)	1 (0.8)	0	1 (3.7)	N.A.
Photophobia, *n* (%)	1 (0.8)	0	1 (3.7)	N.A.
Phonophobia, *n* (%)	1 (0.8)	0	1 (3.7)	N.A.
Balance deficit and ataxia, *n* (%)	1 (0.8)	1 (1.1)	0	N.A.
Hyper/hypotonia, *n* (%)	1 (0.8)	1 (1.1)	0	N.A.
Anxiety disorders, *n* (%)	1 (0.8)	1 (1.1)	0	N.A.
Behavioural changes, *n* (%)	1 (0.8)	1 (1.1)	0	N.A.
Sensibility alterations, *n* (%)	1 (0.8)	1 (1.1)	0	N.A.
Retrograde amnesia, *n* (%)	1 (0.8)	1 (1.1)	0	N.A.
Nistagmus, *n* (%)	1 (0.8)	1 (1.1)	0	N.A.

LOC: loss of consciousness; N.A.: not applicable.

Table S2. Main extra-neurological manifestations in the two subgroups.

Signs and Symptoms	COVID-19 (*n* = 95)	MIS-C (*n* = 27)
Fever, *n* (%)	71 (74.7)	27 (100)
Reduced appetite, *n* (%)	34 (35.8)	5 (18.5)
Respiratory symptoms, *n* (%)	43 (45.3)	12 (44.4)
Cough, *n*	27	4
Rhinitis, *n*	21	1
Respiratory distress, *n*	16	12
Apnea, *n*	3	0
Gastrointestinal symptoms, *n* (%)	25 (26.3)	21 (77.8)
Vomit, *n*	19	14
Diarrhea, *n*	8	12
Abdominal pain, *n*	5	11
Nausea, *n*	4	2
Hepato- and/or splenomegaly, *n* (%)	0	6 (22.2)
Sore throat or pharyngitis, *n* (%)	8 (8.4)	0
Asthenia, *n* (%)	7 (7.4)	8 (29.6)
Arthralgia, *n* (%)	1 (1.1)	3 (11.1)
Artrhitis, *n* (%)	0	1 (3.7)
Myalgia, *n* (%)	2 (2.1)	0
Myositis, *n* (%)	1 (1.1)	0
Otalgia, *n* (%)	3 (3.2)	0
Thoracic pain, *n* (%)	3 (3.2)	2 (7.4)
Palpitations, *n* (%)	1 (1.1)	0
Muco-cutaneous involvement, *n* (%)	5 (5.3)	24 (88.9)
Skin rash, *n*	3	22
Conjunctivitis, *n*	2	19
Mucositis/cheilitis, *n*	0	7
Extremities alterations, *n*	0	2
Uveitis, *n* (%)	0	1 (3.7)
Lymphadenopathy, *n* (%)	0	4 (14.8)
Macrohematuria, *n* (%)	2 (2.1)	0

Table S3. Main laboratory findings in the enrolled patients and in the two subgroups.

**Laboratory Tests**	**Total** (***n*** **= 122)**	**COVID-19** (***n*** **= 95)**	**MIS-C** (***n*** **= 27)**	***p***
Altered CRP/tested pt, *n*/*n* (%)	65/122 (53.3)	38/95 (40)	27/27 (100)	<0.001
CRP, median (IQR) – *mg/dL*	0.73 (0.43-5.37)	0.5 (0.14-1.27)	20.29 (11.25-27.27)	<0.001
Altered PCT/tested pt, *n*/*n* (%)	34/92 (37)	9/65 (13.8)	25/27 (92.6)	<0.001
PCT, median (IQR) – *ng/mL*	0.08 (0.05-2.09)	0.05 (0.05-0.14)	11.03 (2.4-33.8)	<0.001
Altered ESR/tested pt, *n*/*n* (%)	20/36 (55%)	3/16 (18.8)	17/20 (85)	<0.001
ESR, median (IQR) – *mm/h*	44.5 (7.75-69.5)	7.5 (2-26.5)	68 (47.5-91.5)	<0.001
Min WBC count, median (IQR) - c*ell/μL*	6765 (4752.5-9310)	6670 (4815-9175)	7220 (4050-9375)	0.777
Max WBC count, median (IQR) - c*ell/μL*	8825 (6230-16150)	8110 (5690-10850)	18130 (14505-19630)	<0.001
Neutropenia[#]/tested pt, *n*/*n* (%)	36/121 (29.8)	32/94 (34)	4/27 (14.8)	0.060
N min, median (IQR) - c*ell/μL*	2246 (1443-4479)	2033.5 (1294.3-3446.3)	3924 (2303-6507.5)	<0.001
Lymphopenia*/tested pt, *n*/*n* (%)	47/121 (38.8)	27/94 (28.7)	20/27 (74.1)	<0.001
L min, median (IQR) - c*ell/μL*	1910 (995-3545)	2356 (1306.3-4018.3)	823 (499.5-1542)	<0.001
Thrombocytopenia[§]/tested pt, *n*/*n* (%)	17/122 (13.9)	6/95 (6.3)	11/27 (40.7)	<0.001
PLT min, median (IQR) – x10^3 c*ell/μL*	285 (199-388)	314 (216-408)	178 (121-298)	<0.001
Anemia°/tested pt, *n*/*n* (%)	39/122 (32)	17/95 (17.9)	22/27 (81.5)	<0.001
Hb min, mediana (IQR) - *g/dL*	11.5 (10.6-12.7)	11.7 (11-12.8)	10 (8.9-11)	<0.001
Coagulopathy/tested pt, *n*/*n* (%)	11/60 (18.3)	2/33 (6.1)	9/27 (33.3)	<0.001
Fibrinogen, median (IQR) - *mg/dL*	474 (287.5-609)	317 (254.3-431.3)	573 (517.5-674.5)	<0.001
D-dimer, median (IQR) - *μg/L*	2856 (1232.3-5957)	1085 (712.5-1262)	4292.5 (2877-6895.5)	<0.001
T-troponin, median (IQR) - *pg/mL*	30.2 (14.5-96)	22.1 (17.43-36.7)	32 (14.5-193)	0.527
NT-pro-BNP, median (IQR) - *pg/mL*	2547 (581.8-19352)	58 (18-206.5)	9219 (1381.5-26931)	<0.001

CRP: C-reactive protein; ESR: erythrocyte sedimentation rate; Hb: haemoglobin; L: lymphocytes; N: neutrophils; NT-proBNP: amino-terminal pro-hormone brain natriuretic peptide; PCT: procalcitonin; PLT: platelets; pt: patients; WBC: white blood cells

[#]Neutropenia: neutrophils <1.500 cells/μL

*Lymphopenia: lymphocytes <1.500 cells/μL

[§]Thrombocytopenia: platelets <150 x 10^3 cells/μL

°Anemia: haemoglobin < 2 standard deviation of normal range for age

Table S4. Management and outcome in the enrolled patients and in the two subgroups.

Treatment	Total (n = 122)	COVID-19 (n = 95)	MIS-C (n = 27)	p
Antibiotic treatment, n (%)	42 (34.4)	17 (17.9)	25 (92.6)	<0.001
Oral steroids, n (%)	33 (27)	10 (10.5)	23 (85.2)	<0.001
Intravenous steroids, n (%)	30 (24.6)	6 (6.3)	24 (88.9)	<0.001
IVIg, n (%)	30 (24.6)	3 (3.2)	27 (100)	<0.001
Anti-IL1 drugs, n (%)	13 (10.7)	0	13 (48.1)	<0.001
Oxigen/ventilatory support, n (%)	25 (20.5)	12 (12.6)	13 (48.1)	<0.001
Heparin, n (%)	14 (11.5)	2 (2.1)	12 (44.4)	<0.001
Vasoactive/inotropic agents	10 (8.2)	0	10 (37)	<0.001
Anti-SARS-CoV-2 drugs[#], n (%)	1 (0.8)	1 (1.1)	0	N.A.
Monoclonal antibodies[#], n (%)	1 (0.8)	1 (1.1)	0	N.A.
Benzodiazepines*, n (%)	9 (7.4)	8 (8.4)	1 (3.7)	0.682
Lenght of stay, median (IQR) - days	4 (2-9)	3 (2-5)	11 (9-19)	<0.001
ICU admission, n (%)	16 (13.1)	3 (3.2)	13 (48.1)	<0.001
Death, n (%)	0	0	0	N.A.
Symptoms/signs at discharge, n (%)	16 (13.1)	10 (10.5)	6 (22.2)	0.112
Follow-up duration, median (IQR) - days	122 (71.5-197.5)	118 (74.3-171.5)	152 (74.25-355.8)	0.082
Symptoms/signs at last follow-up, n (%)	6 (4.9)	4 (4.2)	2 (7.4)	0.631

ICU: intensive care unit; IL: interleukin; IVIg: intravenous immunoglobulin; N.A.: not applicable.

[#]Remdesivir was the used antiviral drug and Casirivimab and Imdevimab were the used monoclonal antibodies.

*Benzodiazepines were administered by oral, nasal, rectal or parenteral route before the arrive to the hospital, at the Emergency Department or during the ward.
